# Sex differences in cardiovascular function during submaximal exercise in humans

**DOI:** 10.1186/2193-1801-3-445

**Published:** 2014-08-20

**Authors:** Courtney M Wheatley, Eric M Snyder, Bruce D Johnson, Thomas P Olson

**Affiliations:** Division of Cardiovascular Diseases, Mayo Clinic, 200 1st Street, SW, Rochester, MN 55905 USA; Department of Kinesiology, University of Minnesota, Minneapolis, MN USA

**Keywords:** Cardiac output, Arterial pressure, Systemic vascular resistance, Catecholamines, Energy expenditure

## Abstract

Differences in cardiovascular function between sexes have been documented at rest and maximal exercise. The purpose of this study was to examine the sex differences in cardiovascular function during submaximal constant-load exercise, which is not well understood.

Thirty-one male and 33 female subjects completed nine minutes moderate and nine minutes vigorous intensity submaximal exercise (40 and 75% of peak watts determined by maximal exercise test). Measurements included: intra-arterial blood pressure (SBP and DBP), cardiac index (Q_I_), heart rate (HR), oxygen consumption (VO_2_) and arterial catecholamines (epinephrine = EPI and norepinephrine = NE), and blood gases. Mean arterial pressure (MAP), stroke volume index (SV_I_), systemic vascular resistance index (SVR_I_), arterial oxygen content (CaO_2_), arterial to venous O_2_ difference (AVO_2_) and systemic oxygen transport (SOT) were calculated.

At rest and during submaximal exercise Q_I_, SV_I_, SBP, MAP, NE, CaO_2_, and SOT were lower in females compared to males. VO_2_, AVO_2_, EPI were lower in females throughout exercise. When corrected for wattage, females had a higher Q, HR, SV, VO_2_ and AVO_2_ despite lower energy expenditure and higher mechanical efficiency.

This study demonstrates sex differences in the cardiovascular response to constant-load submaximal exercise. Specifically, females presented limitations in cardiac performance in which they are unable to compensate for reductions in stroke volume through increases in HR, potentially a consequence of a female’s blunted sympathetic response and higher vasodilatory state. Females demonstrated greater cardiac work needed to meet the same external work demand, and relied on increased peripheral oxygen extraction, lower energy expenditure and improvements in mechanical efficiency as compensatory mechanisms.

## Background

Control of the cardiovascular system is critical for homeostasis and to respond to stresses such as physical activity (Rowell 1993). During exercise, a number of physiologic adjustments must occur to provide the necessary supply of oxygen and nutrients to meet the demands of muscular work dictated by the task presented (Rowell 1993). As such, the cardiovascular adjustments to exercise have been studied extensively to provide general guidelines regarding the normal or anticipated responses of various physiologic systems.

Numerous physiologic differences exist between men and women including: height, weight, body composition (including fat and lean muscle mass), as well as differences in hormones (e.g. estrogen, progesterone, testosterone, etc.) and hemoglobin levels (Charkoudian and Joyner 2004). With regards to exercise capacity, the enhanced capacity for increased maximal oxygen uptake noted in males is often cited to explain the heightened physical performance in males. However, when individuals are matched for age, height, and lean muscle mass, there remains considerable differences in the cardiovascular response to exercise according to sex, the reasons for which remain less clear (Charkoudian and Joyner 2004). Previous work suggests differences exist in the mechanisms contributing to enhancement of cardiac performance during aerobic exercise between sexes, where females have demonstrated a blunted increase in ejection fraction with exercise (Adams et al. 1987; Higginbotham et al. 1984; Kuo et al. 1987), and unlike men, women demonstrated no increase in stroke volume with exercise training (Spina et al. 1993). Further, women have demonstrated a slower age associated decline in HR reserve with age; in contrast, men have a shown an increase in end diastolic volume index and stroke work index with age (Fleg et al. 1995). Taken together it has been suggested that men have a greater reliance on preload and enhanced use of the Frank-Starling mechanism whereas females rely on heart rate to increase cardiac output. To date, the majority of this previous work has been documented at rest and maximal exercise; leaving a major paucity of data regarding the differences in cardiovascular response to submaximal constant-load exercise between men and women, as findings have primarily been drawn from submaximal workloads during a maximal exercise test (Fleg et al. 1995; Sullivan et al. 1991). As such a number of questions remain unanswered, including: 1) do the reductions in cardiac function demonstrated at peak exercise in females remain at submaximal intensities, 2) what are the underlying mechanisms leading to differences in the cardiovascular response to exercise across sex boundaries, 3) what compensatory mechanisms do females rely on in an attempt to ameliorate limitations, and 4) do the known differences between sexes become more apparent or dissipate when the individuals are non-sedentary (exercising >3 times/week).

Therefore, the purpose of this study was to examine the differences in the cardiovascular response to constant-load submaximal exercise between men and women. To this end, we examined cardiac output and its subcomponents (stroke volume and heart rate) as well as the intra-arterial blood pressure and catecholamine responses to exercise in healthy men and women at rest and during moderate and vigorous intensity constant-load exercise. We hypothesized that despite similar cardiac output during moderate submaximal exercise stroke volume index would be higher in men whereas females would demonstrate higher HR for that given workload. In contrast during vigorous exercise, females would present reduced cardiac output due to an inability to accommodate the added demand via increased HR. Further, we expected males to demonstrate elevated SBP and MAP in comparison to female subjects.

## Results

### Participant characteristics

Table 1 details the demographic characteristics of the participants. Sixty-four healthy participants, male (n = 31) and female (n = 33), were recruited for this study. Although there was no difference in age across sexes (p = 0.44), as expected the males were taller, heavier, and had a higher BMI and BSA compared to the female participants (p < 0.01 for all).

**Table 1:** **Participant characteristics**

	All	Male	Female	p-value
Number	64	31	33	
Age	29.0 ± 0.7	29.6 ± 1.0	28.4 ± 1.1	0.44
Height (m)	1.7 ± 0.0	1.8 ± 0.0	1.7 ± 0.0	<0.001
Weight (kg)	73.0 ± 1.7	82.3 ± 1.8	64.2 ± 1.8	<0.001
BMI (kg/m^2^)	24.1 ± 0.4	25.4 ± 0.6	22.8 ± 0.5	0.002
BSA (m^2^)	1.9 ± 0.0	2.0 ± 0.0	1.7 ± 0.0	<0.001
VO_2_max (mL/min/Kg)	35.0 ± 0.9	39.0 ± 1.3	31.2 ± 1.0	<0.001
VO_2_max (% pred.)	96.1 ± 15.7	95.4 ± 15.5	96.8 ± 16.2	0.73
Peak workload (W)	220.0 ± 7.4	252.1 ± 10.0	189.8 ± 8.1	<0.001

*BMI*, body mass index; *BSA*, body surface area; *VO*_*2*_ *max*, maximal oxygen uptake. Data are presented as mean ± SD. P-value = Students *T*-test male vs. female.

### Sex differences in cardiovascular function at rest

**Figure 1:**
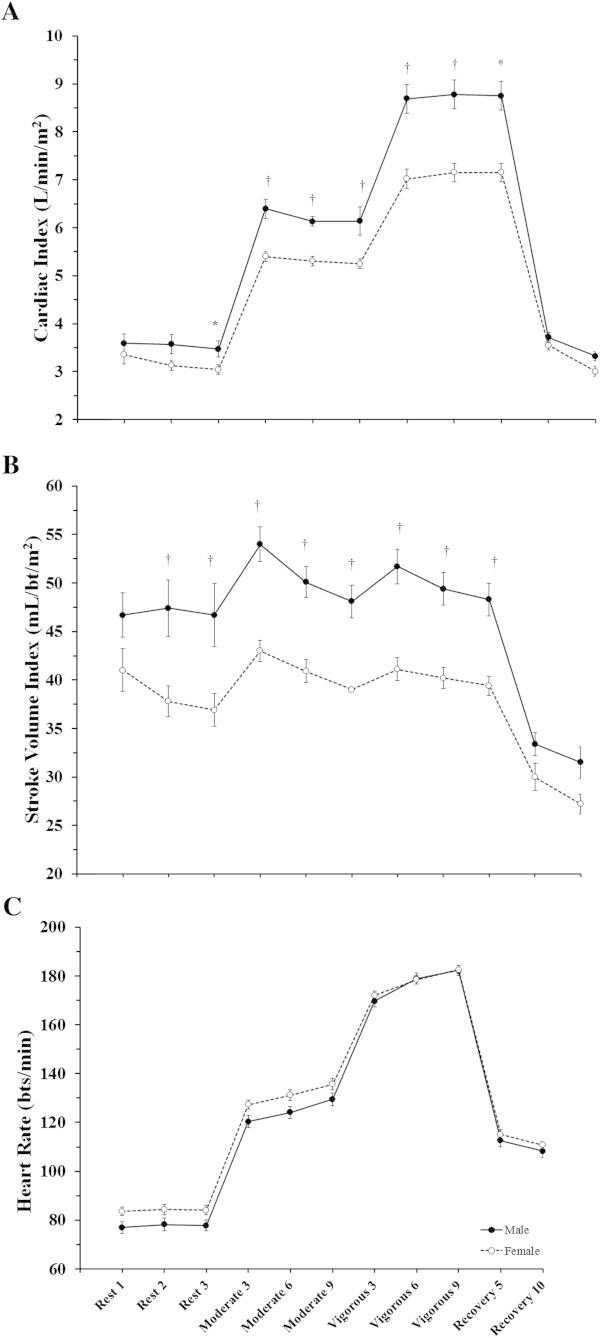
**Sex differences in the cardiac response to constant-load submaximal exercise.** Data are presented as mean ± SEM at rest, moderate intensity constant-load exercise (~40% peak workload), vigorous intensity constant-load exercise (~70% peak workload), and at 5 and 10 minutes of recovery. These data demonstrate the differential cardiac response to constant-load exercise across sexes. Panel **A**: Cardiac Index; Panel **B**: Stroke Volume Index; Panel **C**: Heart Rate. Sex comparison: * = p < 0.05, † = p < 0.01.

**Figure 2:**
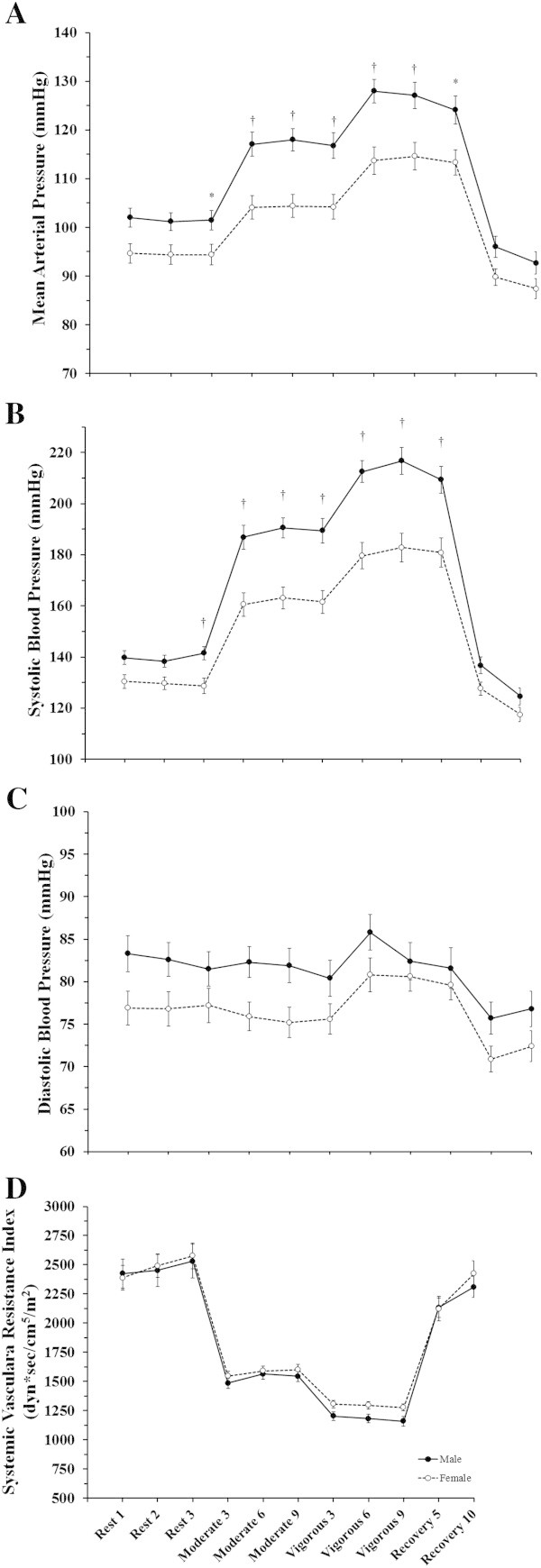
**Sex differences in the systemic hemodynamic response to constant-load submaximal exercise.** Data are presented as mean ± SEM at rest, moderate intensity constant-load exercise (~40% peak workload), vigorous intensity constant-load exercise (~70% peak workload), and at 5 and 10 minutes of recovery. These data demonstrate the differential cardiac response to constant-load exercise across sexes. Panel **A**: Mean Arterial Pressure; Panel **B**: Systolic Blood Pressure; Panel **C**: Diastolic Blood Pressure; Panel **D**: Systemic Vascular Resistance. Sex comparison: * = p < 0.05, † = p < 0.01.

Table 2 highlights the cardiovascular function according to sex during the pre-exercise rest period while individuals were seated upright on the cycle ergometer. At rest there was no difference in VO_2_ (p = 0.44); however, the male participants demonstrated a higher Q_I_ (p = 0.03) mediated by a higher SV_I_ (p = 0.01). There was trend for the female participants to have a higher HR (p = 0.06). The male participants also demonstrated a higher SBP (p < 0.01) with no difference in DBP (p = 0.13). Although the SBP measured via the arterial catheter suggests some of the males may have been mildly hypertensive, the cuff auscultation measurement (118 ± 11) clarifies they were normotensive and the elevated SBP was likely due to the placement of the arterial catheter and that the measurements were taken pre-exercise not in a true resting condition (Wheatley et al. 2013). The higher SBP in the male participants contributed to an elevated MAP (p = 0.02). Since MAP and Q_I_ were both elevated in males, there was no difference in SVR_I_ between the two sexes (p = 0.83). Figures 1 and 2 show data detailing the three measurement time points during the resting period for all cardiovascular measures. Male participants demonstrated greater circulating NE levels (p = 0.03) while EPI levels were similar (p = 0.19) when compared to their female counterparts. Although the partial pressure of oxygen (PaO_2_) was similar between sexes, the lower hemoglobin (Hb) concentration reduced the arterial oxygen content (CaO_2_) and systemic oxygen transport (SOT) in female participants (p < 0.01 for Hb, CaO_2_ and p = 0.02 for SOT). Additionally, the partial pressure of carbon dioxide (PaCO_2_) was higher in the male participants (p = 0.02).

**Table 2:** **Resting physiologic measures**

	All	Male	Female	p-value
**Cardiovascular**				
VO_2_ (mL/min)	274.6 ± 75.8	317.6 ± 76.3	234.1 ± 48.7	<0.01
VCO_2_ (mL/min)	218.2 ± 76.0	259.8 ± 78.6	233.9 ± 47.9	<0.01
VO_2_ (mL/kg/min)	3.75 ± 1.27	3.81 ± 0.83	3.67 ± 0.77	0.44
Q_I_ (L/min/m^2^)	3.2 ± 0.8	3.5 ± 0.9	3.0 ± 0.6	0.03
SV_I_ (mL/m^2^)	41.6 ± 15.3	46.5 ± 18.2	37.1 ± 10.1	0.01
HR (bts/min)	81.0 ± 11.3	77.8 ± 12.3	84.1 ± 9.4	0.06
AVO_2_ Difference (mL O_2_/100 mL blood)	4.7 ± 1.3	4.8 ± 1.4	4.6 ± 1.2	0.52
SBP (mm Hg)	134.9 ± 17.2	141.5 ± 14.5	128.7 ± 17.5	<0.01
DBP (mm Hg)	79.3 ± 11.6	82.0 ± 11.6	76.7 ± 11.2	0.13
MAP (mm Hg)	97.8 ± 12.3	101.8 ± 11.5	94.0 ± 11.9	0.02
SVRI (dyn*sec/cm^5^/m^2^)	255.4 ± 715.2	2544.30. ± 807.1	2564.5.9 ± 629.4	0.83
Hemoglobin (g/dL)	14.4 ± 1.4	15.4 ± 1.2	13.5 ± 0.8	<0.01
SOT (mL/min/m^2^)	624.7 ± 184.1	709.50 ± 212.3	544.2 ± 104.0	0.02
RPE (mL/min/m^2^)	6.4 ± 0.0	6.1 ± 0.4	6.0 ± 0.0	0.31
**Blood gases**				
PaO_2_ (mm Hg)	100.7 ± 11.7	102.3 ± 11.2	99.0 ± 10.9	0.25
PaCO_2_ (mm Hg)	35.1 ± 4.2	35.5 ± 4.9	34.8 ± 3.5	0.02
CaO_2_ (mL/100 mL)	19.14 ± 1.8	20.4 ± 1.6	17.9 ± 1.1	<0.01
**Catecholamines & lactate**				
Epinephrine (pg/mL)	95.8 ± 53.5	104.8 ± 38.3	87.3 ± 64.1	0.19
Norepinephrine (pg/mL)	297.9 ± 86.7	322.7 ± 93.8	274.7 ± 73.6	0.03
Lactate (mM)	0.77 ± 0.37	0.78 ± 0.26	0.75 ± 0.45	0.68

VO_2_, volume of oxygen consumption; Q_I_, cardiac index; SV_I_, stroke volume index; HR, heart rate; AVO_2_ difference, arterial to venous O_2_ difference; SBP, systolic blood pressure (direct arterial); DBP, diastolic blood pressure (direct arterial); MAP, mean arterial pressure (direct arterial); SVR_I_, systematic vascular resistance index; SOT, systematic oxygen transport; RPE, rating of perceived exertion; PaO2, arterial partial pressure of oxygen; PaCO_2_, arterial partial pressure of carbon dioxide; CaO_2_, arterial oxygen content. Data are presented as mean ± SD for 3 measurement timepoints during the resting period. P-value = Students *T*-test male vs. female.

### Sex differences in cardiovascular function during moderate intensity constant-load exercise

**Figure 3:**
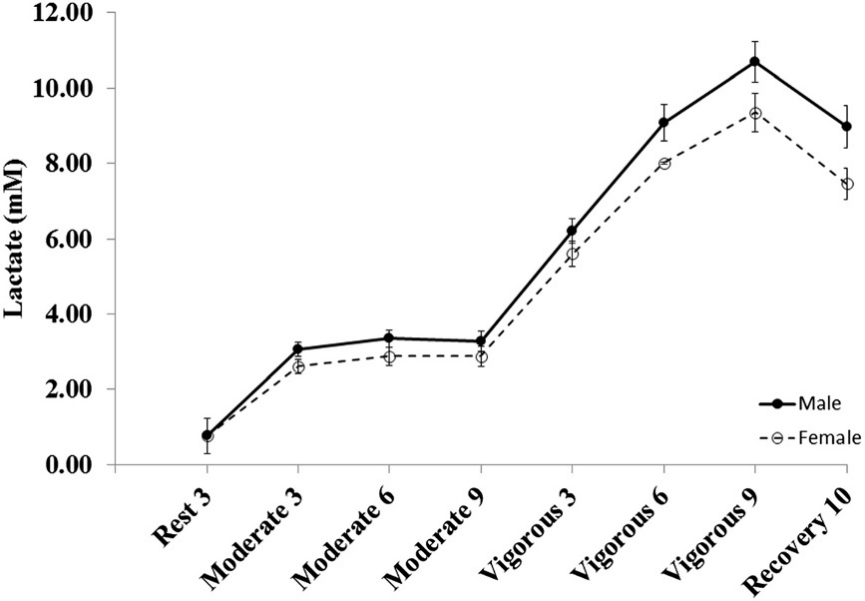
**Lactate response to constant-load submaximal exercise.** Data are presented as mean ± SEM at the last minute of rest, moderate intensity constant-load exercise (~40% peak workload), vigorous intensity constant-load exercise (~70% peak workload), and 10 minutes of recovery.

Table 3 describes the cardiovascular response to moderate intensity steady-state exercise among males and females. The male participants demonstrated a higher VO_2_ (p < 0.01) with an elevated Q_I_ (p < 0.01) mediated by a higher SV_I_ (p < 0.01). During moderate intensity exercise, the reduced oxygen carrying capacity, due to lower Hb levels (p < 0.01) in female participants was sufficient to result in lower oxygen consumption and contributed to a reduced SOT mediated by a lower CaO_2_ and Q_I_. Similar to at rest, the PaCO_2_ was higher in the male participants (p < 0.01). The female participants also demonstrated a trend for higher HR (p = 0.07), although this difference was not large enough to compensate for the lower SV_I_ and subsequently reduced Q_I_ in females. The male participants demonstrated a higher SBP (p < 0.01) which contributed to higher MAP (p < 0.01) as there was no difference in DBP between males and females (p = 0.11). As was seen at rest, the differential response between males and females in Q_I_ and MAP did not result in a difference in SVR_I_ between sexes (p = 0.41). Both NE and EPI levels were higher in the male participants (p < 0.01). Blood lactate increased from baseline in both groups and stabilized over the nine minute bout. Figures 1 and 2 detail data collected for all three measurement time points the moderate intensity constant-load exercise period for all cardiovascular measures, blood lactate measured for all three time points is provided in Figure 3.

**Table 3:** **Cardiovascular response to moderate intensity submaximal steady-state exercise**

	All	Male	Female	p-value
**Cardiovascular**				
VO_2_ (mL/min)	1284.0 ± 43.0	1545.7 ± 48.1	1038.2 ± 33.7	<0.01
VCO_2_ (mL/min)	1161.7 ± 39.4	1403.3 ± 43.4	934.7 ± 30.9	<0.01
VO_2_ (mL/kg/min)	17.7 ± 3.0	19.0 ± 3.3	16.5 ± 2.0	<0.01
Q_I_ (L/min/m^2^)	5.8 ± 0.8	6.2 ± 0.8	5.4 ± 0.6	<0.01
SV_I_ (mL/m^2^)	45.8 ± 9.1	50.4 ± 9.2	41.3 ± 6.4	<0.01
HR (mL/min)	128.1 ± 13.2	124.6 ± 13.7	131.4 ± 12.1	0.04
AVO_2_ Difference (mL O_2_/100 mL blood)	11.9 ± 1.4	12.6 ± 1.2	11.2 ± 1.2	<0.01
SBP (mm Hg)	174.9 ± 27.5	188.8 ± 23.5	161.8 ± 24.7	<0.01
DBP (mm Hg)	78.5 ± 10.1	82.2 ± 10.2	74.9 ± 8.6	0.01
MAP (mm Hg)	110.6 ± 14.7	117.7 ± 13.2	103.9 ± 13.0	<0.01
SVR_I_ (dyn*sec/cm^5^/m^2^)	1556.1 ± 233.2	1574.4 ± 243.8	1564.3 ± 225.3	0.72
Hemoglobin (gh/dL)	15.4 ± 1.4	16.2 ± 1.1	14.0 ± 0.7	<0.01
SOT (mL/min/m^2^)	1150.1 ± 226.3	1315.7 ± 187.6	994.5 ± 127.7	<0.01
RPE	10.4 ± 1.3	10.4 ± 1.4	10.4 ± 1.1	0.58
**Blood gases**				
PaO_2_ (mm Hg)	97.7 ± 7.1	97.2 ± 6.0	98.1 ± 8.0	0.56
PaCO_2_ (mm Hg)	37.5 ± 2.7	38.4 ± 2.6	36.6 ± 2.4	<0.01
CaO_2_ (mL/100 mL)	19.9 ± 1.8	21.3 ± 1.4	18.5 ± 0.9	<0.01
**Catecholamines & lactate**				
Epinephrine (pg/mL)	100.8 ± 37.9	113.2 ± 35.3	89.1 ± 37.1	0.01
Norepinephrine (pg/mL)	630.1 ± 163.3	693.2 ± 172.4	570.88 ± 130.8	0.02
Lactate (mM)	3.00 ± 1.269	3.22 ± 1.24	2.78 ± 1.32	0.17
Lactate fold change from baseline	3.3 ± 2.0	3.4 ± 2.1	3.1 ± 1.9	0.52

See Table 2 for acronyms explained.

### Sex differences in cardiovascular function during vigorous intensity constant-load exercise

Table 4 details the differences in cardiovascular function at vigorous intensity constant-load exercise between sexes. Despite being at a matched relative workload, the male participants demonstrated a higher VO_2_ (p < 0.01). The male participants also demonstrated a higher Q_I_ (p < 0.01) mediated by a higher SV_I_ (p < 0.01) since there was no difference between the groups for HR (p = 0.95). CaO_2_ and SOT again remained lower and likely contributed to the lower VO_2_ in female participants (p < 0.01). Again, PaCO_2_ was higher in the male participants (p < 0.01). Similar to the moderate intensity workload, the male participants demonstrated a higher SBP (p < 0.01) which contributed to a higher MAP (p < 0.01) since there was again no difference in DBP (p = 0.51). In contrast to the lack of difference at the moderate intensity workload, the male participants demonstrated a lower SVR_I_ compared to the female participants (p = 0.04). Figures 1 and 2 show data for all 3 measurement time points during the vigorous intensity constant-load exercise for all cardiovascular measures. Blood lactate continued to accumulate until the end of exercise in both sexes, but tended to be lower in females (Figure 3).

**Table 4:** **Cardiovascular response to vigorous intensity submaximal steady-state exercise**

	All	Male	Female	p-value
**Cardiovascular**				
VO_2_ (mL/min)	2167.2 ± 664.2	2685.0 ± 504.9	1664.0 ± 335.5	<0.01
VCO_2_ (mL/min)	2257.7 ± 744.5	2801.8 ± 603.7	1746.4 ± 440.7	<0.01
VO_2_ (mL/kg/min)	29.5 ± 5.9	32.9 ± 5.7	26.1 ± 3.9	<0.01
Q_I_ (L/min/m^2^)	7.9 ± 1.4	8.7 ± 1.4	7.1 ± 0.9	<0.01
SV_I_ (mL/m^2^)	44.7 ± 9.2	49.6 ± 9.5	40.1 ± 6.3	<0.01
HR (bts/min)	177.2 ± 11.0	176.7 ± 12.4	177.7 ± 9.9	0.95
AVO_2_ Difference (mL O_2_/100 mL blood)	14.4 ± 1.6	15.3 ± 1.3	13.6 ± 1.4	<0.01
SBP (mm Hg)	196.2 ± 32.5	212.9 ± 26.3	180.1 ± 30.4	<0.01
DBP (mm Hg)	81.9 ± 10.6	83.5 ± 11.6	80.1 ± 9.5	0.29
MAP (mm Hg)	120.0 ± 15.8	126.6 ± 140.	113.4 ± 14.9	<0.01
SVR_I_ (dyn*sec/cm^5^/m^2^)	1237.8 ± 192.9	1190.4 ± 200.0	1279.4 ± 180.9	0.047
Hemoglobin (g/dL)	15.5 ± 1.5	16.7 ± 1.1	14.4 ± 0.7	<001
SOT (mL/min/m^2^)	1619.9 ± 371.1	1889.5 ± 303.5	1366.6 ± 219.4	<0.01
RPE	16.4 ± 0.9	16.4 ± 0.9	16.3 ± 0.9	0.75
**Blood gases**				
PaO_2_ (mm Hg)	98.6 ± 7.4	97.6 ± 6.9	99.4 ± 7.7	0.17
PaCO_2_ (mm Hg)	32.9 ± 2.9	33.6 ± 2.8	32.4 ± 2.9	0.18
CaO_2_ (mL/100 mL)	20.4 ± 1.9	21.9 ± 1.5	19.1 ± 0.9	<0.01
**Catecholamines & lactate**				
Epinephrine (pg/mL)	318.0 ± 231.3	389.7 ± 286.2	250.7 ± 137.7	0.01
Norepinephrine (pg/mL)	2252.8 ± 859.5	2508. ± 946.3	2012.5 ± 701.5	0.02
Lactate (mM)	8.16 ± 2.46	8.79 ± 2.39	7.68 ± 2.39	0.08
Lactate fold change from baseline	11.1 ± 5.1	11.5 ± 5.9	10.9 ± 4.3	0.61

See Table 2 for acronyms explained.

### Sex differences in cardiovascular function during constant-load exercise correcting for differences in peak power

Due to the apparent difference in aerobic fitness between sexes (i.e. the women’s VO_2MAX_ was 80% that of the men’s maximal aerobic capacity) and the likely differences in muscle mass, we corrected for differences in cardiovascular parameters dictated by maximal power output. Although the relative intensities were the same between sexes, 40 and 70% of maximal workload, we also compared responses as a function of the absolute power (wattage) performed by each subject (Table 5). When corrected for watts, absolute VO_2_ was not different between groups, demonstrating that the relative intensity, both moderate and vigorous, were similar between groups. During both moderate and vigorous intensity constant-load exercise, the female subjects appeared to be at a higher cardiopulmonary demand, as VO_2_ (ml/kg/min), SV, HR, Q and AVO_2_ difference corrected for watts were all higher when compared to their male counterparts (p < 0.01, for all). Work and energy expenditure were significantly higher in males at both moderate and vigorous workloads (Table 6). When correcting for differences in wattage, there was no difference at moderate intensity, but the energy expenditure per watt was lower in females at the vigorous intensity. There was no difference in mechanical efficiency at the moderate intensity (23% for both) between sexes, but at the vigorous workload females were slightly more efficient (24 vs 23% females vs. males respectively), and the delta efficiency resulted in females being slightly more efficient with increasing intensity whereas there was no change in the males (Table 6). During both exercise intensities, the ventilatory equivalent for O_2_ and CO_2_ was higher in females when corrected for watts (Table 6).

**Table 5:** **Cardiovascular function during constant-load exercise correcting for differences in peak power**

	All	Male	Female	p-value
**Moderate**				
VO_2_ (mL/min)/Watt	16.24 ± 2.00	15.96 ± 2.01	16.5 ± 1.99	0.26
VO_2_ (mL/kg/min)/Watt	0.23 ± 0.05	0.20 ± 0.03	0.26 ± 0.05	<0.01
Q (L/min)/Watt	0.14 ± 0.02	0.13 ± 0.02	0.15 ± 0.03	<0.01
SV (mL)/Watt	1.0 ± 0.18	1.05 ± 0.13	1.14 ± 0.21	<0.01
HR (bts/min)/Watt	1.77 ± 0.65	1.34 ± 0.38	2.18 ± 0.58	<0.01
AVO_2_ Difference (mL O_2_/100 mL blood)/Watt	0.19 ± 0.23	0.19 ± 0.34	0.18 ± 0.04	<0.01
**Vigorous**				
VO_2_ (mL/min)/Watt	13.75 ± 0.94	13.85 ± 0.84	13.65 ± 1.03	0.4
VO_2_ (mL/kg/min)/Watt	0.20 ± 0.04	0.17 ± 0.02	0.22 ± 0.04	<0.01
Q (L/min)/Watt	0.10 ± 0.01	0.09 ± 0.01	0.10 ± 0.01	<0.01
SV (mL)/Watt	0.55 ± 0.08	0.52 ± 0.06	0.57 ± 0.09	<0.01
HR (bts/min)/Watt	1.24 ± 0.40	0.95 ± 0.25	1.50 ± 0.38	<0.01
AVO_2_ Difference (mL O_2_/100 mL blood)/Watt	0.10 ± 0.03	0.08 ± 0.02	0.11 ± 0.02	<0.01

See Table 2 for acronyms explained.

**Table 6:** **Work, energy expenditure and mechanical efficiency during moderate and vigorous constant-load exercise**

	All	Male	Female	p-value
Resting energy expenditure (kcal/min)	1.31 ± 0.36	1.52 ± 0.36	1.01 ± 0.24	<0.01
**Moderate**				
Work (kcal/min)	1.15 ± 0.36	1.41 ± 0.29	0.92 ± 0.22	<0.01
Energy expenditure (kcal/min)	5.01 ± 1.50	6.13 ± 1.14	3.96 ± 0.91	<0.01
Energy expenditure/Watts (kcal/min)/Watt	0.62 ± 0.006	0.063 ± 0.009	0.63 ± 0.01	0.52
V_E_/VO2	27.5 ± 3.4	26.1 ± 2.7	28.9 ± 3.4	<0.01
V_E_/VO_2_/Watt	0.38 ± 0.15	0.28 ± 0.08	0.48 ± 0.13	<0.01
V_E_/VCO2	30.5 ± 2.8	29.0 ± 1.9	32.0 ± 2.8	<0.01
Mechanical efficiency (%)	23.2 ± 3.7	23.1 ± 3.8	23.4 ± 3.6	0.59
**Vigorous**				
Work (kcal/min)	2.29 ± 0.73	2.81 ± 0.60	1.80 ± 0.46	<0.01
Energy expenditure (kcal/min)	9.59 ± 3.19	11.98 ± 2.45	7.37 ± 1.96	<0.01
Energy expenditure/Watts (kcal/min)/Watt	0.006 ± 0.007	0.061 ± 0.006	0.059 ± 0.007	<0.01
V_E_/VO2	34.4 ± 4.9	33.4 ± 5.0	35.3 ± 4.6	<0.01
V_E_/VO_2_/Watt	0.24 ± 0.09	0.18 ± 0.06	0.30 ± 0.08	<0.01
V_E_/VCO2	32.8 ± 4.2	31.5 ± 4.0	34.0 ± 4.0	<0.01
Mechanical efficiency (%)	24.1 ± 2.6	23.5 ± 2.3	24.6 ± 2.7	<0.01
Delta efficiency (vigorous-moderate)	0.82 ± 2.12	0.37 ± 2.25	1.24 ± 1.69	0.10

## Discussion

This study examined the influence of sex on the cardiovascular response to submaximal steady-state exercise in healthy humans. The results of this study suggest that during steady-state exercise at matched relative workloads of both moderate (40% peak workload) and vigorous (75% peak workload) submaximal intensity the female participants demonstrated lower Q mediated by reduced SV compared to males even when indexed for BSA. In addition, the female participants demonstrated lower SBP (with non-significantly lower DBP) resulting in reduced MAP. Despite these differences, the SVR remained similar at rest and moderate intensity and only became different during vigorous intensity exercise between the male and female participants suggesting that the regulation and homeostatic relationship between blood pressure and cardiac output is achieved through different mechanisms in males and females. This research advances our current understanding of the differences in cardiovascular hemodynamics during exercise between sexes for a number of reasons including the recruitment of a robust sample size, placement of an intra-arterial and a venous catheter for measures of arterial blood pressure and simultaneous assessment of plasma catecholamines and blood gases. Further, we have analyzed our data to extensively consider differences in body size and work capacity which will affect cardiovascular responses independent other sex-mediated differences.

When comparing the Q_I_ and SV_I_ across sexes to account for differences in body size, our results suggest that both the male and female participants had a similar HR, with a trend for higher HRs in females, at both submaximal exercise intensities, consistent with prior studies evaluating across relative workloads during a maximal exercise test (Ogawa et al. 1992; Fleg et al. 1995). Despite this similarity, Q_I_ was lower at both exercise intensities in female participants as a result of reduced SV_I_. This would suggest that since Q_I_ was lower and HR was similar between the groups, the female participants had a greater reliance on HR to increase Q_I_ compared to the male participants. This follows the conclusions by Ogawa et al. that differences in stroke volume are the primary cause of sex differences in cardiac output during maximal and now submaximal exercise (Ogawa et al. 1992). Our work extends upon the previous work by both Fleg et al. and Sullivan et al. which examined the sex-based differences in cardiac volumes and cardiac index in subjects <40 years old group during peak upright cycle exercise (Fleg et al. 1995; Sullivan et al. 1991). In contrast to our findings, neither of these previous studies noted any differences in Q_I_ and SV_I_ between sexes at relative submaximal exercise. Although all groups were of the same age, there were clear differences in fitness level as these other populations were sedentary, excluding anyone who exercise >3 times/week. The fitness/activity level difference between their populations and ours is clear when reviewing the lower peak work rates in both sexes when compared to our population (men: 149/157 vs. 252 W; females: 102/115 vs. 190 W, subject populations: Fleg/Sullivan vs Wheatley respectively). We believe the ability of our subjects to reach higher workloads and create a greater cardiac demand, allowed us to see differences that were not apparent in the sedentary individuals of the previous work. Additionally, the methodology used to assess cardiac function differed between the current and previous studies, in that the previous studies utilized gated radionuclide angiography for assessment of cardiac volumes: end diastolic and systolic volumes, ejection fraction, and direct Fick for Q and SV, whereas we measured Q from the acetylene open circuit method. The open circuit acetylene method for measurement of Q has been validated against direct Fick and shown to have a strong correlation particularly during exercise. In some instances, Q measured via acetylene open circuit may underestimate actual Q at higher workloads suggesting that the differences we present in the current study may have been larger if calculated from direct Fick (Johnson et al. 2000a).

Despite exercising subjects at the same percentage of peak watts to keep subjects at similar relative exercise intensity, VO_2_ remained different between sexes at both submaximal intensities. Because VO_2_ and Q follow wattage, we also made comparisons after correcting for differences in wattage to determine if this was driving the differences observed. We found that the differences remained between males and females except that they were in the opposite direction, such that for a given wattage females were demanding more, having a greater Q and its components, SV and HR, for a given wattage when compared to male participants. This elevated Q, HR, and SV highlights a greater cardiac work needed to meet the same physical work demands. In an attempt to compensate for the specific reduced cardiac performance, these women increased peripheral oxygen extraction as evidenced by the higher AVO_2_ difference when corrected for wattage. Analysis of mechanical work, energy expenditure, and efficiency also suggest the tendency to be more efficient with their resources especially during vigorous exercise which allowed females to accommodate the increase in cardiopulmonary demand. Additionally, lactate accumulation tended to be lower in females at the end of vigorous exercise following the rightward displacement of lactate accumulation in response to maximal exercise in women which has been demonstrated previously and suggested to provide a potential offset for their lower VO_2_ and greater cardiac demand by allowing for increased capacity to accomplish the work (Sargent and Scroop 2007).

This study extends upon previous investigation conducted during maximal exercise and demonstrates that during submaximal constant-load exercise intensities men have a greater reliance on the Frank-Starling mechanism to meet the increased cardiac demand and that the differences in cardiac output are driven by limitations in stroke volume in females. Previous research suggests a reason for this apparent blunted increase in stroke volume may be differences autonomic regulation in that females have reduced sympathetic and α-adrenergic responsiveness and a greater reliance on β-receptor mediated vasodilatation and increased parasympathetic tone (Kneale et al. 1997; Kneale et al. 2000; Schmitt et al. 2010; Evans et al. 2001). Further, although measurements were taken at rest, muscle sympathetic nerve activity (MSNA), a measure of sympathetic system activity, has been shown to be lower in females (Charkoudian et al. 2005). Additionally, the lower incidence of cardiovascular disease in premenopause women and marked increase in the incidence of hypertension around the age of menopause has been attributed to changes in female reproductive hormones where estrogen has been shown to reduce NE-induced vasoconstriction, can promote endothelium-dependent vasodilation through increasing the bioavailability of NO, and increase β_2_-receptor sensitivity (Charkoudian 2001; Kneale et al. 1997; Hart et al. 2011; Sudhir et al. 1997). Both Fleg et al. and our current study demonstrate lower NE in females at rest and during exercise, which could limit β_1_-receptor activation and thereby reduce cardiac contractility. Therefore, these data suggest that females demonstrate reduced or limited SV and subsequently Q possibly due blunted sympathetic nervous system activity along with an elevated basal vasodilatory state limiting/blunting the increase in vascular tone to augment venous return and sympathetic activity to increase cardiac contractility during exercise. To this regard, it appears that the females in this study attempted to compensate for this through increasing HR (via increased parasympathetic withdrawal) and oxygen extraction. This increase in extraction is in line with previous work where arterial O_2_ content has been shown to be reduced in females solely due to differences in Hb as arterial O_2_ was similar, but mixed venous O_2_ was lower at rest and during submaximal exercise when compared to men (Sullivan et al. 1991).

A critical component of the cardiovascular response to exercise is an increase in blood pressure to maintain perfusion pressure to the heart, brain, and other vital organs (Rowell 1993). Changes in blood pressure are a function of the change in cardiac output and the peripheral resistance to forward flow typically mediated by vascular tone. Despite a significant increase in sympathetically mediated vasoconstriction, typically peripheral resistance will fall during exercise due greater metabolically mediated vasodilation of the active muscles (Joyner 2006; Joyner et al. 2008). However, in most cases, blood pressure will increase since the increase in cardiac output is more than adequate to compensate for the reduction in peripheral resistance.

It is known that in young healthy men, total peripheral resistance is positively related to MSNA, suggesting that a primary mediator of vascular tone is sympathetic nervous system outflow (Narkiewicz et al. 2005). Interestingly, it has been shown that in young healthy women, resting blood pressure is typically lower than that observed in men of the same age (Burt et al. 1995). Moreover, the incidence of orthostatic hypotension is greater in women than in men (Fu et al. 2005), and women have lower tonic autonomic support of resting arterial pressure (Christou et al. 2005). These observations would suggest that the contribution of sympathetically mediated vasoconstrictor tone to arterial pressure regulation differs in men and women. In fact, it has recently been demonstrated that typically the interrelationships between SVR, Q, and sympathetic neural control of vascular tone do not exist in young women under resting conditions (Hart et al. 2009). Specifically, Hart and colleagues suggest that in young healthy women, sympathetic nerve activity does not determine SVR and further Q is not balanced with sympathetic activity to maintain normal arterial pressure as is typically seen in young healthy men (Hart et al. 2009).

Our results demonstrate that the female participants had a lower resting blood pressure compared to the male participants. Moreover, at both levels of submaximal exercise the female participants demonstrated significantly lower systolic blood pressure which contributed to lower MAP. However, these differences coupled with the differences in cardiac output resulted in a similar SVR response to the exercise session, following what has been demonstrated in relative stages of peak exercise testing previously (Fleg et al. 1995; Ogawa et al. 1992). This suggests that although blood pressure was ultimately lower in the female participants, the SVR response was the same between the male and female participants.

### Limitations

In this study percentage of peak work rate was used to match relative intensities between sexes, however, recent work has suggested this methodology is not ideal due to differences in gas exchange threshold and critical power between individuals (Katch et al. 1978; Lansley et al. 2011). As such the moderate and vigorous intensities compared in this study may under/overestimate work rates or physiological demand for all individuals, which could shift individuals to different points on the VO_2_ kinetic continuum and thereby alter the cardiopulmonary demand. Although using % work rate to match exercise intensities may increase the variability of the responses, our lactate measurements taken every three minutes during these submaximal workloads suggests that the intensities were quite similar for the majority of the individuals and between sexes. The blood lactate levels increased and plateaued during the moderate intensity work, and increased at each measurement through the nine minutes the vigorous intensity work as would be expected for both sexes. However, continued research in this area is needed to validate our findings, particularly at submaximal intensities. One alternative approach to standardizing cardiac demand may be to use the percent delta concept as described by Lansley et al. (Lansley et al. 2011)*.* Additionally, females in this study were not all tested in the early follicular phase of their menstrual cycle which could have added additional variability to the response observed in these women; however controlling for this phase would likely have made the differences noted in this study more robust. Additional insight may be gleaned with future research examining females in both their early follicular phase and during their luteal phase.

## Conclusion

The results of this study suggest a differential regulation of cardiovascular function between sexes during constant-load submaximal exercise, corroborating with previous findings of differences in cardiovascular function at rest and maximal exercise. Correcting for differences in size, females demonstrate a lower SV, which they are unable to compensate for through increases in HR as Q_I_ and SV_I_ are both lower in females. When corrected for wattage, females demonstrate elevated Q, HR, and SV with increased peripheral oxygen extraction as a compensatory mechanism (indicated by higher VO_2_ and AVO_2_ difference and improvements in mechanical efficiency) when compared to males highlighting greater cardiac work needed to meet the same physical work demand. The limitations in stroke volume could be the result of a female’s blunted sympathetic response and higher basal vasodilatory state, evidenced by lower catecholamine levels in women at rest and through submaximal exercise. Moreover both sexes had similar SVR while SBP and MAP were higher in men suggesting that the relationship between Q and MAP remained similar across sexes. These data are in agreement with previous findings suggesting sex differences in the regulation of arterial tone and extend these findings to suggest that the outcome of the difference in regulation is similar (i.e. no difference in SVR).

## Methods

### Participant characteristics

Sixty-four healthy adults (men: 21–40 years; women: 20–40 years), non-sedentary (exercising >3 times/week) were recruited to participant in this study. All participants were recruited from the surrounding community and were healthy, non-smokers with no evidence of cardiovascular, pulmonary, or musculoskeletal disease and were not pregnant or currently taking any medications (except for birth control). The protocol was approved by the Mayo Clinic Institutional Review Board and all participants gave written informed consent prior to participation. All aspects of the study conformed to the Declaration of Helsinki and Health Insurance Portability and Accountability Act (HIPAA) guidelines.

### Overview of protocol

Parts of the data generated by this study have been published elsewhere, where the objective was to explore the cardiovascular consequences of exercise according to beta-2 adrenergic receptor genotype (Snyder et al. 2006) and to examine the difference between manual cuff auscultation vs. intra-arterial blood pressure measurement techniques (Wheatley et al. 2013). In these studies, data was grouped according to beta-2 adrenergic receptor genotypes or as a whole comparing agreement between the blood pressure measurement techniques within an individual. The cardiovascular parameters in these previous manuscripts were presented under different stratification and with a different focus, but never evaluating differences in cardiovascular response between sexes as presented in this manuscript.

During visit one, participants reported to the Mayo Clinic – Clinical Research Unit to provide a blood sample to rule out anemia and pregnancy in women. All of the participants underwent baseline pulmonary function testing according to ATS standards (ATS 1995) (Elite Series Plethysmography, Medical Graphics, St. Paul, MN) and an incremental exercise test on an electromagnetically braked upright cycle ergometer to maximal volition (Corival, Lode Medical Technology, Netherlands). The incremental exercise test progressed in a manor appropriate for the subject’s body size and his/her reported type, speed, and intensity of exercise training to provide a test duration of 10–16 minutes or five to eight stages (test duration: males 14:00 ± 2:47; females 13:07 ± 2:21, p = 0.10). Subjects exercised at an initial workload ranging between 20–50 watts (mean initial workload: males 36 ± 8 W; females 25 ± 7 W) with the workload increasing by this initial workload (wattage) every two minutes until exhaustion.

On visit two, participants were instrumented with a 5 cm 20-gauge Teflon coated indwelling catheter (FA-04020, Arrow International Inc., Reading, PA) placed in the radial artery after local anesthesia with 2% lidocaine. This catheter was used for the direct measurement of arterial blood pressure (BP). Resting measurements of Q, heart rate (HR), and BP were made and stroke volume (SV) was calculated. The participants then exercised for nine minutes at ~40% (moderate intensity) and nine minutes at ~75% (vigorous intensity) of their peak workload achieved during the initial exercise studies while measurements were repeated every 2–3 minutes. Nine-minutes of exercise at each workload was performed because pilot data in our laboratory suggested that this was an adequate time frame to obtain three sets of measurements, and bringing the participants close to exhaustion during the higher workload.

For both study visits, prior to exercise testing participants were fitted with a nose clip and a mouthpiece attached to a PreVent Pneumotach (Medical Graphic, St. Paul, MN). For maximal exercise testing, all participants were verbally encouraged to continue the exercise protocol to maximal volition, determined by an inability to maintain a cadence between 60 to 80 rpm, a respiratory exchange ratio greater than 1.10 and/or a rating of perceived exertion (RPE) ≥17 (Borg scale = 6-20) (Borg 1982). This baseline exercise study served as a screening tool and was used to determine work intensities for study visit two.

### Data collection

#### Measurement of ventilation and gas exchange

Oxygen consumption (VO_2_), carbon dioxide production (VCO_2_), minute ventilation, respiratory rate and tidal volume were measured continuously during all exercise testing using a metabolic measurement system (MedGraphics CPX/D; Medical Graphics, St. Paul, MN) interfaced with a mass spectrometer (MGA 1100, Marquette Electronics, Milwaukee, WI). Manual volume calibration was performed with a three liter syringe and gas calibration was performed using manufacturer-recommended gases of known concentration. All calibration procedures were conducted immediately prior to each testing protocol. This system has been validated against classic “Douglas bag” collection techniques, and the stability of measurements verified by regular testing at standard exercise intensities by laboratory personnel (Proctor and Beck 1996).

#### Assessment of cardiovascular function

Cardiac output was measured using a 8–10 breath open-circuit acetylene wash-in technique as described previously (Johnson et al. 2000b). Briefly, the pneumotachograph was connected to a non-rebreathing Y valve (Hans Rudolph, KC, MO) with the inspiratory port connected to a pneumatic switching valve (Hans Rudolph, KC, MO) which allowed for rapid switching from room air to the test gas mixture (a large reservoir containing 0.7%C_2_H_2_, 21%0_2_, 9%He, Balance N_2_). Gases were sampled using the mass spectrometer which was integrated with custom analysis software for the assessment of Q using our previously described iterative technique which has been validated against the direct Fick method (Johnson et al. 2000b). Heart rate was measured continuously by standard 12-lead electrocardiogram (GE Case, GE Medical Systems, Milwaukee, WI). Stroke volume was calculated by dividing the Q by the HR. Both Q and SV were standardized to body surface area (BSA) to obtain the Q and SV indexes (Q_I_ and SV_I_, respectively).

Intra-arterial BP was measured using a SpaceLab 512D patient monitor (SpaceLabs Inc., Hillsboro, OR). Mean arterial pressure (MAP) was calculated using the equation: MAP = DBP + 1/3(SBP-DBP), where DBP is diastolic blood pressure and SBP is systolic blood pressure. Systemic vascular resistance (SVR) was calculated as the mean arterial pressure divided by Q and multiplied by 80 (conversion from Woods units). Systemic vascular resistance was then divided by BSA to obtain the systemic vascular resistance index (SVR_I_). The intra-arterial catheter also provided access for collection of arterial blood for the measurement of arterial catecholamines (see below for catecholamines), arterial blood gases, lactate, and hemoglobin content (partial pressure of arterial oxygen (PaO_2_) and oxygen saturation (SaO_2_). Arterial oxygen content (CaO_2_) was calculated using the following equation:


Systemic oxygen transport (SOT) was calculated as CaO_2_ multiplied by Q.

Arterial-venous oxygen (AVO_2_) difference was calculated according to the Fick equation:


#### Calculation of work, energy expenditure and mechanical efficiency

Work was converted from watts to kcal/min by multiplying watts by 0.01433. Energy expenditure (EE) was calculated by subtracting resting VO_2_ (mL/min) from the exercising VO_2_ and multiplying by the RER caloric equivalent taken from the table published by Foss and Keteyian (Foss and Keteyian 1988; p 83). Mechanical efficiency was then determined by dividing work by energy expenditure and expressing as a percentage.

#### Measurement of catecholamines and lactate

Epinephrine (EPI), norepinephrine (NE) and lactate were measured according to methods developed in the Mayo Clinic Clinical Research Unit immunochemical core laboratory and the methods of Sealey (Sealey 1991). For EPI, our laboratory intra-assay coefficients of variation (CVs) are 12.2% and 3.6% at 13.8 and 242 pg/mL. Inter-assay CVs are 8.5% and 6.3% at 179 and 390 pg/mL. Plasma lactate was measured using Roche Cobas c311 chemistry analyzer using an enzymatic reaction to convert lactate to pyruvate (Roche Diagnostics Corp. Indianapolis, IN 46250). Intra-assay CVs are 8.9% and 1.6% at 0.8 and 4.9 mmol/L respectively. Inter-assay CVs are 4.9%, and 1.2% at 1.2 and 4.6 mmol/L respectively.

### Statistical analysis

Statistical analysis and graphic presentation were accomplished using SPSS (v 12.0, Chicago, IL) and GraphPad Prizm (v 4.0, San Diego, CA). The demographic data was examined using two-tailed unpaired Students T-tests. Analysis of variance (ANOVA) with Tukey HSD was conducted to determine differences at rest and during exercise for Q, HR, SV, SBP, DBP, MAP and SVR between groups. All data were found to have homogeneity of variance prior to the ANOVA using Levene’s test for equality of variance. Statistical significance was set at an alpha level of 0.05 for all analyses. Data are presented as mean ± standard deviation unless otherwise indicated.

## Abbreviations

AVO_2_: Arterial to venous O2 difference
BMI: Body mass index
BSA: Body surface area
CaO_2_: Arterial oxygen content
DBP: Diastolic blood pressure
EPI: Epinephrine
HR: Heart rate
MAP: Mean arterial blood pressure
NE: Norepinephrine
PaCO_2_: Arterial partial pressure of carbon dioxide
PaO_2_: Arterial partial pressure of oxygen
Q: Cardiac output
Q_I_: Cardiac index
RPE: Rating of perceived exertion
SaO_2_: Oxygen saturation
SBP: Systolic blood pressure
SOT: Systemic oxygen transport
SV: Stroke volume
SV_I_: Stroke volume index
SVR: Systemic vascular resistance
SVR_I_: Systemic vascular resistance index
VCO_2_: Carbon dioxide production
VO_2_: Oxygen consumption.
